# Comparative Analysis of Paper-Based and Web-Based Versions of the National Comprehensive Cancer Network-Functional Assessment of Cancer Therapy-Breast Cancer Symptom Index (NFBSI-16) Questionnaire in Breast Cancer Patients: Randomized Crossover Study

**DOI:** 10.2196/18269

**Published:** 2021-03-02

**Authors:** Jinfei Ma, Zihao Zou, Emmanuel Eric Pazo, Salissou Moutari, Ye Liu, Feng Jin

**Affiliations:** 1 Department of Breast Surgery The First Affiliated Hospital of China Medical University Shenyang China; 2 Department of Ophthalmology He Eye Hospital Shenyang China; 3 Mathematical Science Research Centre Queen's University Belfast Belfast United Kingdom

**Keywords:** breast cancer, NFBSI-16, patient-reported outcome, reproducibility, test-retest reliability, web-based questionnaire

## Abstract

**Background:**

Breast cancer remains the most common neoplasm diagnosed among women in China and globally. Health-related questionnaire assessments in research and clinical oncology settings have gained prominence. The National Comprehensive Cancer Network–Functional Assessment of Cancer Therapy–Breast Cancer Symptom Index (NFBSI-16) is a rapid and powerful tool to help evaluate disease- or treatment-related symptoms, both physical and emotional, in patients with breast cancer for clinical and research purposes. Prevalence of individual smartphones provides a potential web-based approach to administrating the questionnaire; however, the reliability of the NFBSI-16 in electronic format has not been assessed.

**Objective:**

This study aimed to assess the reliability of a web-based NFBSI-16 questionnaire in breast cancer patients undergoing systematic treatment with a prospective open-label randomized crossover study design.

**Methods:**

We recruited random patients with breast cancer under systematic treatment from the central hospital registry to complete both paper- and web-based versions of the questionnaires. Both versions of the questionnaires were self-assessed. Patients were randomly assigned to group A (paper-based first and web-based second) or group B (web-based first and paper-based second). A total of 354 patients were included in the analysis (group A: n=177, group B: n=177). Descriptive sociodemographic characteristics, reliability and agreement rates for single items, subscales, and total score were analyzed using the Wilcoxon test. The Lin concordance correlation coefficient (CCC) and Spearman and Kendall τ rank correlations were used to assess test-retest reliability.

**Results:**

Test-retest reliability measured with CCCs was 0.94 for the total NFBSI-16 score. Significant correlations (Spearman ρ) were documented for all 4 subscales—Disease-Related Symptoms Subscale–Physical (ρ=0.93), Disease-Related Symptoms Subscale–Emotional (ρ=0.85), Treatment Side Effects Subscale (ρ=0.95), and Function and Well-Being Subscale (ρ=0.91)—and total NFBSI-16 score (ρ=0.94). Mean differences of the test and retest were all close to zero (≤0.06). The parallel test-retest reliability of subscales with the Wilcoxon test comparing individual items found GP3 (item 5) to be significantly different (*P*=.02). A majority of the participants in this study (255/354, 72.0%) preferred the web-based over the paper-based version.

**Conclusions:**

The web-based version of the NFBSI-16 questionnaire is an excellent tool for monitoring individual breast cancer patients under treatment, with the majority of participants preferring it over the paper-based version.

## Introduction

Breast cancer accounts for the highest proportion of malignant tumors among women (excluding skin cancers) globally. According to an International Agency for Research on Cancer report [1], the worldwide burden for breast cancer was 2.1 million cases in the year 2018, accounting for 1 in 4 cancer cases among women. Advancements in breast cancer screening, detection, and treatment over the last few decades have produced an increased chance of cure for early-stage breast cancer patients, while advanced (metastatic) disease patients now have prolonged survival and varying degrees of controlled symptoms [2,3]. However, full-aspect and long-term treatment can impact patients’ and survivors’ quality of life and therefore require continual health management during and after the process of recovery [4].

Breast cancer and its treatment have been documented to significantly disrupt patients’ health-related quality of life, which has been found to predict survival time and additionally showed more significance for noncurative patients [5-10]. To assess treatment benefits, patient-reported outcome measures (PROMs) provide unique perspectives on cancer symptoms from patients’ experience, some of which can be neglected by clinicians and laboratory tests [11-13]. The National Comprehensive Cancer Network–Functional Assessment of Cancer Therapy–Breast Cancer Symptom Index (NFBSI-16) PROMs were regulated on the foundation of the Functional Assessment of Chronic Illness Therapy (FACIT) measurement system to assess high-priority symptoms of breast cancer, emphasizing patients’ input, which can be applied to help evaluate the effectiveness of treatments for breast cancer in clinical practice and research [14-16].

The migration from paper-based to web-based versions does not guarantee preservation of psychometric properties of the scale since various factors have the potential to impact the performance of the questionnaire scale when adapted for web-based administration, such as layout, instructions, or restructuring of item and response. Researchers have investigated methods of validation, routes of administration, practical considerations, and reliability of electronic PROMs [17-27]. Gwaltney et al’s meta-analysis on assessing the equivalence of computer versus paper versions of PROMs showed “a high overall level of agreement between paper and computerized measures” [28]. The review encompassed the fields of rheumatology, cardiology, psychiatry, asthma, alcoholism, pain assessment, gastrointestinal disease, diabetes, and allergies. In contrast, a study of the European Organization for Research and Treatment of Cancer Quality of Life Questionnaire-Core 30 found small but statistically significant differences in scale mean scores (3 to 7 points on a 100-point scale) associated with mode of administration [29]. Various validated web-based questionnaires in oncology have been demonstrated to be reliable and effective tools for assessing PROMs in therapeutic clinical and research settings [30-33]. Currently in China, web-based versions of clinical research questionnaires using WeChat are rapidly growing in number, and various studies have validated the WeChat-based administration of health-related questionnaires [34-36]. To cover the large growing patient base in China, we expected web-based administration of the NFBSI-16 to be a reliable methodology to assess the impact of disease, treatment, and well-being status among patients with breast cancer. Additionally, it could be a more cost-effective and efficient method to apply in the growing number of patients in certain demographics.

The aim of this study was to analyze the reliability of a web-based NFBSI-16 questionnaire (Chinese language) for measuring disease- and treatment-related symptoms and concerns in breast cancer patients, comparing it with the validated paper-based version.

## Methods

### Study Design and Patient Enrolment

Patients were recruited from the Department of Breast Surgery of the First Affiliated Hospital of China Medical University, Shenyang, China, between October 2019 and January 2020. The inclusion criteria were female gender, full legal age, proven diagnosis of breast cancer, being under systemic anticancer treatment, ability to follow study instructions, sufficient literacy and fluency in Chinese to comprehend the questionnaires, ability and willingness to complete the study protocol, and signed declaration of consent. Potential participants were excluded if they could not provide informed consent or participated in other studies (burden of participation). Participants had an initial clinic visit at which eligibility was assessed. All eligible participants were randomly chosen from the hospital’s central registry and invited to volunteer for the study via face-to-face interview with a trained research clinician. Written informed consents were obtained. The study protocol was approved by the First Affiliated Hospital of China Medical University ethics committee.

The study was a randomized crossover design in which all participants completed both a standard paper questionnaire and a web-based version of the NFBSI-16 (Multimedia Appendix 1). Patients in group A were assigned to start with the paper-based version followed by the web-based version on their smartphone in the same session. Patients in group B completed the web-based version followed by the paper-based version. Participants were randomized immediately after enrolment to group A or B in a 1:1 ratio using a computer-generated randomization list with a specified seed and block size of 6, based on the mode of administration to be completed first. Between each session from paper-based to web-based and web-based to paper-based, participants were given a break of 15 minutes during which they were invited into a quick patient education seminar, which was also a routine activity in our department as a distractor task to lower the potential carryover effect. All participants were provided with written instructions for completion of the paper- or web-based questionnaires prior to their questionnaires being administered. After completing both versions of the NFBSI-16 questionnaire, participants were invited to state their preference for either the paper- or web-based NFBSI-16 questionnaire.

### Questionnaire

The NFBSI-16 contains items from the original FBSI and FACIT measures selected by patients and clinicians according to their priority concern [15], which presented as a more direct tool to reflect the effectiveness of treatments for advanced breast cancer. The NFBSI-16 comprises 16 items with 4 dimensions for ease of use and scoring: Disease-Related Symptoms Subscale–Physical (DRS-P), Disease-Related Symptoms Subscale–Emotional (DRS-E), Treatment Side Effects Subscale (TSE), and Function and Well-Being Subscale (FWB). Therefore, clinicians and researchers can individually view and assess subscale scores when concerned about a particular class of symptoms. The questionnaires were self-completed, and careful attention was paid to the design and layout of the web-based version. In order to reduce the risk of errors in posing, interpretation, recording, and coding responses and potential interrater variability, the theory-based guidelines for self-administered questionnaire design were followed by the authors (Multimedia Appendix 1) [37]. The web-based user interface and paper for the paper-based questionnaires were free from all other information such as logos, slogans, advertisement, etc. The instructions for completing the web-based and paper-based questionnaires were included at the beginning of the web-based interface and header of the paper, respectively. In brief, while participating in the web-based assessment, patients had to scan a redesignated Quick Response code using their smartphone. This action automatically took them to a web-based test, and the user had to select the intensity or severity of the 16 items. After completing the 16th question, the interface turned into a blank screen indicating the test was over. On the other hand, the paper-based questionnaire test was conducted using white paper and pencil. The text was printed using clear 12-point font.

### Testing of the Instrument

During pretesting and pilot testing, 3 colleagues specializing in oncology and 3 nonexperts evaluated the web-based questionnaire’s usability, accessibility, and clarity of the user interface. This testing was only conducted on the functionality of the web-based questionnaire since the format, structure, and sequence of items in the web-based questionnaire were the same as in the validated paper-based questionnaire.

### Computation of Subscale Scores

Data from the paper questionnaires were entered manually into an electronic patient management system by the authors, and data from the web-based questionnaires were automatically captured after the participant completed the online questionnaire and downloaded to the electronic patient management system. All data was anonymized. We assessed the completeness of the data on a per-item basis and questionnaire basis. The total scores were obtained by taking the mean score across completed items and multiplying by 16, the number of items (following official guidelines) [15]. All subscale totals ranged from 0 to 4, with a score of 0 representing that the patient agrees with the item “not at all” and 4 representing “very much”. Subscale scores and total scores were computed for each participant and each mode of administration separately. Comparative analyses of individual items, subscales, and total score were the primary goal of the study.

### Statistical Analysis

All statistical analyses were conducted using SPSS Statistics, version 25 (IBM Corp). Frequency analysis was performed to determine the descriptive sociodemographic characteristics of patients. Referring to ISPOR ePRO Good Research Practices Task Force recommendations [21], we conducted the evaluation of measurement equivalence. Reliability, internal consistency, disparity of responses, and the rate of consistency between paper- and web-based responses were assessed. Reliability was calculated for the 16 individual items as well as for scores of the 4 subscales (DRS-P, DRS-E, TSE, and FWB) and the NFBSI-16 total score in accordance with the NFBSI-16 guidelines [15]. The primary study outcome was to assess the reliability of single items and total score of the web-based questionnaire. The Wilcoxon test was used to identify possible statistically significant differences in the test of parallel forms reliability, both between the single items and the scores due to the ordinal nature of the data. The secondary outcome measure was to assess the consistency and agreement of the web-based questionnaire with the paper-based questionnaire. The mean values of the paper- and web-based measures were calculated, consistency analyses were performed by calculation of the Spearman rank correlation coefficient (Spearman ρ), and agreement rates for each item were assessed using rank correlation (Kendall τ) for each scale. As a second measure of test-retest reliability, we calculated the Lin concordance correlation coefficient (CCC) [38]. Finally, all answers to the ‘‘preference’’ questionnaire were compared between the web-based and the paper version of the NFBSI-16 using χ^2^ tests. In all analyses, *P*<.05 (2-tailed) was considered indicative of statistically significant differences (α=.05). As such an analysis is considered an explorative study, all reported *P* values can be taken as purely descriptive. All figures (box plot and correlation diagram) were generated in SPSS Statistics.

## Results

### Enrolment of Patients

**Figure 1:**
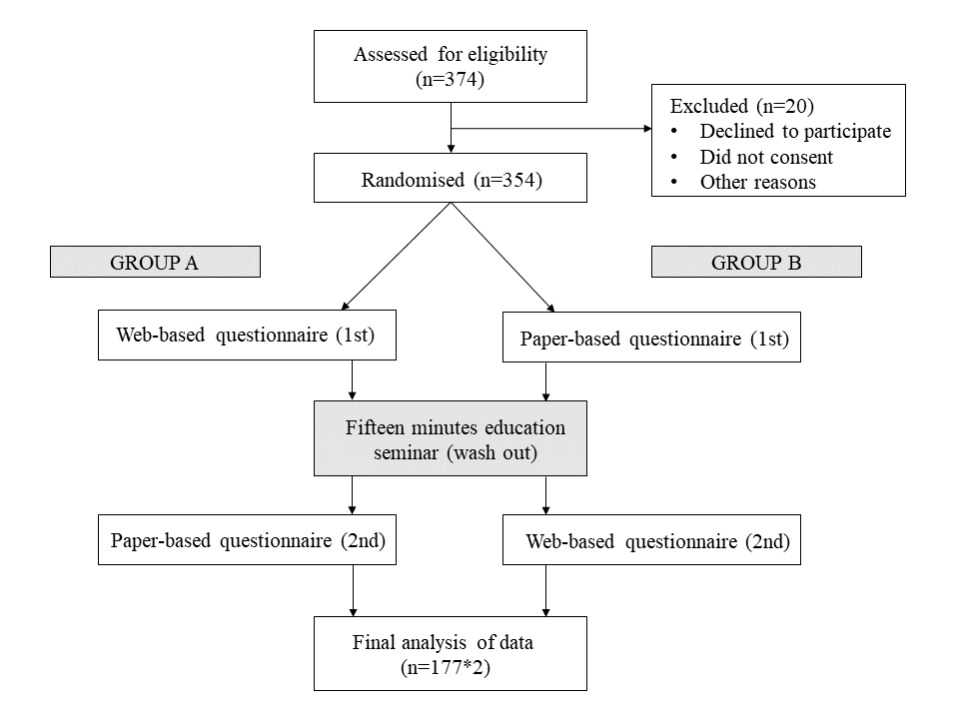
Study flow diagram.

The final analysis included 354 patients with breast cancer receiving systematic treatment who completed both the paper- and web-based versions of NFBSI-16 questionnaire. Initially, 380 patients were assessed for eligibility. 26 patients were excluded, as shown in the study flow diagram (Figure 1). Since there was no internal difference between group A and group B, demographically, two groups were combined in the final analysis. The mean age was 49.5 years (SD 10.44). Other basic characteristics of patients are shown in Table 1.

**Table 1:** Basic characteristics of study participants.

Patient characteristics		n (%)
**Menstrual status**		
	Premenopause	133 (37.6)
	Perimenopause	107 (30.2)
	Postmenopause	114 (32.2)
**Level of education completed**		
	Primary	59 (16.7)
	Secondary	161 (45.5)
	Tertiary	134 (37.9)
**Marital status**		
	Single	16 (4.5)
	Married	338 (95.5)
**Region**		
	Rural	165 (46.6)
	Urban	189 (53.4)
**Treatment**		
	Neoadjuvant therapy	244 (68.9)
	Adjuvant therapy	110 (31.1)

### Parallel Forms Reliability

**Figure 2:**
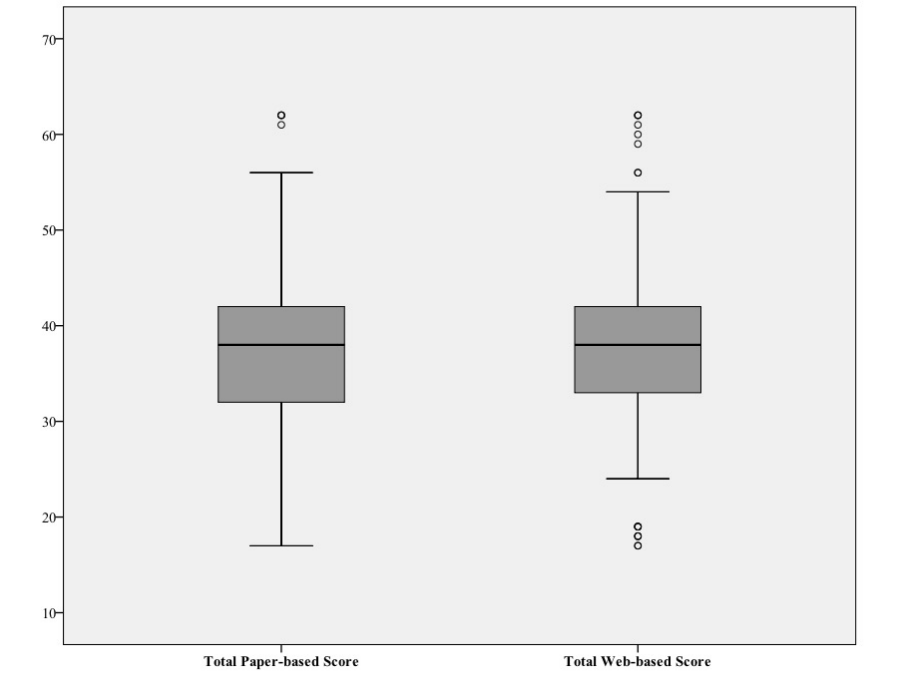
Box plot comparison of paper-based and web-based distribution of total scores.

The Wilcoxon signed rank test analyzed parallel reliability in the single items of the NFBSI-16, shown in Table 2. No systematic location difference between the two versions of questionnaires (paper- and web-based versions) was observed for continuous variables except for item 5 (GP3 question). A very large proportion of the items answered by the patients had the same response (ties) in both versions of the questionnaire, suggesting high parallel reliability as only one significant difference (out of 16 in total) could be found in the single-item comparison. A statistically significant difference could only be identified in question GP3, “Because of my physical condition, I have trouble meeting the needs of my family.” GP3 was reported slightly higher in the paper-based questionnaire (mean 2.07, SD 0.98), while in the web-based version the same participants scored it at a mean of 2.00 (SD 0.91). Additionally, the medians of the item GP3 for the paper- and web-based questionnaires were the same (median 2; IQR 1-3). While the web-based total mean score was slightly higher than the paper-based score by 0.08 points, they had no statistically significant difference between them. Figure 2 illustrates the distribution of the paper-based and web-based total scores in a box plot. The slightly higher total web-based total score can be attributed to a few outliers shown in the box plot. The web-based whisker of the box plot IQR was within the broader IQR of the paper-based version. In addition, slight differences of less than 0.50 points were found between the paper-based and web-based questionnaires when the item scores of the 4 dimensions (DRS-P, DRS-E, TSE, and FWB) were calculated and compared. However, all 4 dimensions’ scores showed no statistically significant differences when compared (Table 3).

**Table 2:** Parallel test-retest reliability of single items and total score (Wilcoxon test).

NFBSI-16^a^ items		Paper-based patient score		Web-based patient score		*P* value	Δ |Mean−Mean'|
		Mean (SD)	Median (IQR)	Mean' (SD)	Median (IQR)		
**Disease-Related Symptoms Subscale – Physical (DRS-P)**							
	GP1 (item 1)	2.31 (0.92)	2 (2-3)	2.32 (0.90)	2 (2-3)	.58	0.01
	GP4 (item 2)	2.19 (0.90)	2 (2-3)	2.17 (0.88)	2 (2-3)	.36	0.02
	GP6 (item 3)	2.29 (1.13)	2 (1-3)	2.30 (1.15)	2 (1-3)	.88	0.01
	B1 (item 4)	2.01 (0.89)	2 (1-3)	2.00 (0.88)	2 (1-3)	.79	0.01
	GP3 (item 5)	2.07 (0.98)	2 (1-3)	2.00 (0.91)	2 (1-3)	.02^b^	0.07
	HI7 (item 6)	2.59 (1.02)	2 (2-3)	2.59 (1.06)	2 (2-3)	.91	0.00
	BP1 (item 7)	1.88 (0.93)	2 (1-2)	1.90 (0.93)	2 (1-2)	.27	0.02
	GF5 (item 8)	2.59 (1.18)	2 (2-3)	2.55 (1.17)	2 (2-3)	.38	0.04
**Disease-Related Symptoms Subscale – Emotional (DRS-E)**							
	GE6 (item 9)	2.00 (1.04)	2 (1-2)	2.01 (1.05)	2 (1-2)	.73	0.01
**Treatment Side Effects Subscale (TSE)**							
	GP2 (item 10)	2.20 (1.15)	2 (1-3)	2.25 (1.10)	2 (1-3)	.16	0.05
	N6 (item 11)	1.87 (0.98)	2 (1-2)	1.85 (0.93)	2 (1-2)	.42	0.02
	GP5 (item 12)	2.77 (1.01)	3(2-3)	2.75 (1.00)	3 (2-3)	.45	0.02
	B5 (item 13)	2.98 (1.35)	3 (2-4)	2.98 (1.33)	3 (2-4)	.89	0.00
**Function and Well-Being Subscale (FWB)**							
	GF1 (item 14)	2.52 (1.04)	2 (2-3)	2.55 (1.01)	2.5 (2-3)	.14	0.03
	GF3 (item 15)	2.82 (1.12)	3 (2-4)	2.81 (1.08)	3 (2-4)	.83	0.01
	GF7 (item 16)	2.82 (1.19)	3 (2-4)	2.85 (1.21)	3 (2-4)	.67	0.03
**Total score**							
	NFBSI-16 score	37.92 (7.79)	38 (32-42.5)	37.88 (7.71)	38 (32.75-42)	.98	0.04

^a^NFBSI-16: National Comprehensive Cancer Network–Functional Assessment of Cancer Therapy–Breast Cancer Symptom Index.

^b^Statistically significant difference.

**Table 3:** Parallel test-retest reliability of subscales (Wilcoxon test).

NFBSI-16^a^ subscale	Paper-based patient outcome		Web-based patient outcome		*P* value	Δ |Mean−Mean'|
	Mean (SD)	Median (IQR)	Mean (SD)	Median (IQR)		
Disease-Related Symptoms Subscale–Physical	35.90 (9.59)	36 (30-42)	35.64 (9.58)	34 (10-42)	.43	0.26
Disease-Related Symptoms Subscale–Emotional	32.00 (16.54)	32 (16-32)	32.05 (16.84)	32 (16-32)	.98	0.05
Treatment Side Effects Subscale	39.20 (13.39)	40 (28-48)	39.20 (12.86)	36 (32-48)	.62	0.00
Function and Well-Being Subscale	43.37 (13.37)	42.67 (32-53.33)	43.62 (13.72)	42.67 (32-53.33)	.32	0.25

^a^NFBSI-16: National Comprehensive Cancer Network–Functional Assessment of Cancer Therapy–Breast Cancer Symptom Index.

### Test of Internal Consistency

**Figure 3:**
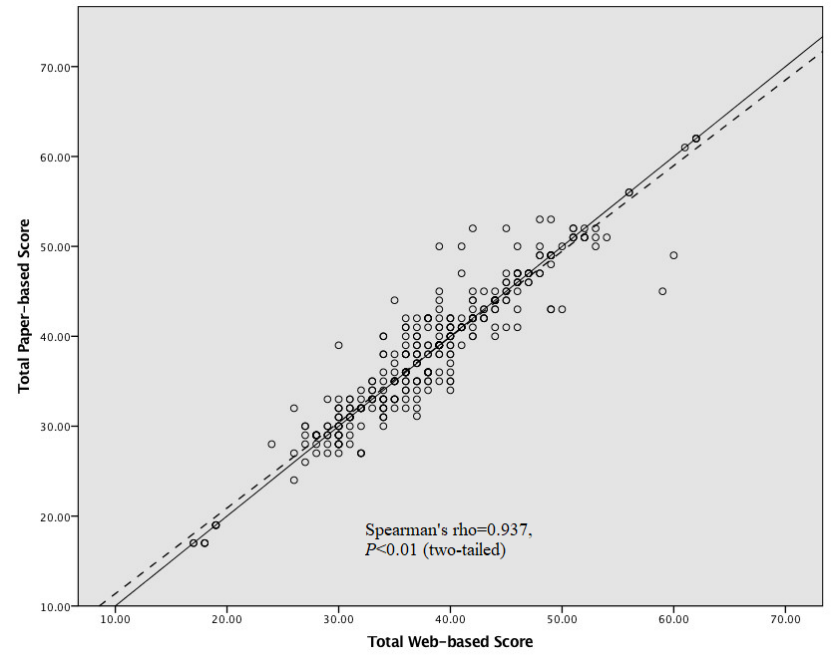
Correlation between total paper-based and web-based scores.

Table 4 shows the Spearman ρ correlation values between the individual items from the paper- and web-based questionnaires. All 16 items demonstrated a high correlation (>0.8) between paper- and web-based items. Individual item internal consistency test was performed by Kendall τ analysis between the two versions. In all items, the rank correlation was high as the Kendall τ coefficients ranged between 0.787 and 0.877 and were all statistically significant. With each data point reflecting an individual patient’s total NFBSI-16 score, Figure 3 depicts a positive correlation between total paper-based and web-based scores. Overall, CCC agreement between paper-based and web-based questionnaires’ item scores were all comparably high at 0.94 (fair: 0.21-0.40; moderate: 0.41-0.60; substantial: 0.61-0.80; almost perfect: 0.81-1.00), as represented in Table 5.

**Table 4:** Correlation between test-retest in individual items and subscale (Spearman ρ and Kendall τ analysis).

Items		Spearman ρ	*P* value	Kendall τ	*P* value
**Disease-Related Symptoms Subscale – Physical (DRS-P)**					
	GP1 (item 1)	0.89	<.001	0.877	<.001
	GP4 (item 2)	0.84	<.001	0.810	<.001
	GP6 (item 3)	0.86	<.001	0.804	<.001
	B1 (item 4)	0.90	<.001	0.87	<.001
	GP3 (item 5)	0.85	<.001	0.825	<.001
	HI7 (item 6)	0.85	<.001	0.813	<.001
	BP1 (item 7)	0.89	<.001	0.856	<.001
	GF5 (item 8)	0.84	<.001	0.796	<.001
	Subscale total	0.93	<.001	0.827	<.001
**Disease-Related Symptoms Subscale – Emotional (DRS-E)**					
	GE6 (item 9)	0.85	<.001	0.826	<.001
	Subscale total	0.85	<.001	0.882	<.001
**Treatment Side Effects Subscale (TSE)**					
	GP2 (item 10)	0.88	<.001	0.830	<.001
	N6 (item 11)	0.89	<.001	0.857	<.001
	GP5 (item 12)	0.83	<.001	0.795	<.001
	B5 (item 13)	0.84	<.001	0.788	<.001
	Subscale total	0.95	<.001	0.882	<.001
**Function and Well-Being Subscale (FWB)**					
	GF1 (item 14)	0.82	<.001	0.787	<.001
	GF3 (item 15)	0.86	<.001	0.821	<.001
	GF7 (item 16)	0.83	<.001	0.79	<.001
	Subscale total	0.91	<.001	0.825	<.001
**Total score**					
	Score	0.94	<.001	0.823	<.001

**Table 5:** Agreement between paper-based and web-based questionnaires scores (Lin concordance correlation coefficient analysis).

Items		R_c_^a^	95% CI
**Disease-Related Symptoms Subscale – Physical (DRS-P)**			
	GP1 (item 1)	0.92	0.90-0.94
	GP4 (item 2)	0.85	0.82-0.88
	GP6 (item 3)	0.86	0.83-0.88
	B1 (item 4)	0.9	0.88-0.71
	GP3 (item 5)	0.86	0.83-0.89
	HI7 (item 6)	0.86	0.83-0.89
	BP1 (item 7)	0.88	0.87-0.91
	GF5 (item 8)	0.85	0.82-0.88
	Subscale total	0.94	0.93-0.95
**Disease-Related Symptoms Subscale – Emotional (DRS-E)**			
	GE6 (item 9)	0.84	0.81-0.87
	Subscale total	0.84	0.81-0.87
**Treatment Side Effects Subscale (TSE)**			
	GP2 (item 10)	0.87	0.85-0.90
	N6 (item 11)	0.88	0.86-0.91
	GP5 (item 12)	0.86	0.83-0.89
	B5 (item 13)	0.83	0.80-0.86
	Subscale total	0.96	0.95-0.97
**Function and Well-Being Subscale (FWB)**			
	GF1 (item 14)	0.85	0.82-0.88
	GF3 (item 15)	0.86	0.83-0.89
	GF7 (item 16)	0.84	0.81-0.87
	Subscale total	0.91	0.89-0.93
**Total score**			
	Score	0.94	0.93-0.95

^a^R_c_: concordance correlation coefficient.

### Patient Preference

Table 6 shows a majority of the participants preferred answering the same questions in a web-based format rather than paper-based format. The difference in preference was statistically different.

**Table 6:** Analysis of participant preference.

Patient preference	Observed, n	Expected, n	Residual	Chi-square (*df*)	Asymptotic significance
Preferred paper-based questionnaire	98	177	−79		
Preferred web-based questionnaire	256	177	79		
Total	354			70.5^a^ (1)	.001^b^

^a^0 cells (0.0%) have expected frequencies less than 5. The minimum expected cell frequency is 177.0.

^b^Statistically significant difference.

### Estimation of the Carryover Effect

To assess the carryover effect, we let *s_A_* denote the sum (total scores from web-based items plus the total scores from paper-based items for each respondent) from group A and let *s_B_* denote the sum from group B. We estimate the carryover effect in both groups (A and B) using the Wilcoxon test on the sum values *s_A_* and *s_B_*, and at a level of significance of 5%, the possible carryover effect is not significantly different between the different sequences (*P*=0.84).

## Discussion

### Principal Results

Overall, reliability was considered to be excellent for the web-based version as measured with the Wilcoxon signed rank test and CCC. Additionally, Spearman ρ correlation and Kendall τ analysis showed that mean differences were all close to zero, supporting good reliability of the web-based version of the NFBSI-16 self-administered questionnaire. In this study, we used the Wilcoxon signed rank test and CCC to assess test reliability. However, different methods can be used to assess test-retest reliability, and there is much discussion in the literature on the best possible methodology [39]. Intraclass correlation coefficient (ICC) was first introduced in 1954 and is a modification of the Pearson correlation coefficient. However, modern ICC is calculated by mean squares (ie, estimates of the population variances based on the variability among a given set of measures) obtained through analysis of variance (ANOVA). The disadvantage of ICC in patient group analysis is that if the groups are mainly homogeneous, the ICC tends to be low, because the ICC compares variance among patients to total variance. If patient groups are mainly heterogeneous, the ICC tends to be high. Thus, ICC would only generalize to similar populations. Additionally, the 1-way ICC does not consider the order in which observations were made [40]. Therefore, the CCC is a useful measure as it not only covers mean differences between the first and second measurements, such as ICCs calculated by a 1-way ANOVA, but also takes the variance differences between the first (paper-based) and second (web-based) measurements into consideration by reducing the magnitude of the resulting test-retest reliability estimate. In conclusion, CCC is a better tool that distinguishes bias between imprecision [39,40].

### Limitations

This study may also have some limitations. First, the significant difference in item 5 (GP3) between paper- and web-based measurement of the NFBSI-16 (Table 3) was an unexpected finding. We think this significant difference might be due to an outlier. This assumption was supported by the fact that even though 293 out of 354 (total) patients had the same answer for the paper- and web-based for item 5 (high number of similarities), a significant difference in the mean was detected. Second, according to the nature of this study, it is difficult to generalize some of our findings as its limited by demographic settings.

### Comparison With Prior Work

NFBSI-16 includes all 8 items from the original FBSI and 8 additional items from FACIT measures, which cover most essential breast cancer–related symptoms and concerns endorsed by both oncology patients and clinicians [15]. Compared to the previous version (FBSI), it emphasizes patient input following Food and Drug Administration guidance for PROMs [41] and has been validated as a comprehensive and powerful tool to evaluate the effectiveness of treatments for breast cancer in clinical practice and research. In addition, the layout of 4 clear separated subscales benefits any clinicians, patients, or researchers by allowing them to view particular domains they are concerned about. However, the reliability of an electronic version in Chinese language has not been tested. This paper describes the evaluation of the test-retest reliability of the web-based version of the NFBSI-16 self-administered questionnaire. When designing a web-based version of a validated paper-based questionnaire, one has to take into consideration variables such as text size, column formatting, contrast, layout, use of corrective lenses, etc. We created the web-based NFBSI-16 to be consistent with the original as far as possible. In addition, technology skills required to complete a web-based questionnaire can differ from those needed to complete a paper-based questionnaire. However, our study found no clinically significant differences between scores obtained from the paper-and web-based versions. Gwaltney et al’s [28] meta-analysis reported the average correlation between paper-based and electronic assessment was 0.90 (95% CI 0.87-0.92; n=32). Our findings suggest that the NFBSI-16 questionnaire achieved a good test-retest reliability, with the total NFBSI-16 score correlation equal to 0.94.

### Conclusions

In summary, the web-based version of the NFBSI-16 clearly showed comparable reliability and is thus a promising measure in evaluating studies in patients undergoing treatment for breast cancer and in monitoring individuals. The test-retest reliability supports the value of the web-based version of the NFBSI-16 for clinical studies with relatively moderate sample sizes. Furthermore, the majority of participants in our study preferred it over the paper-based version; we recommend using the web-based version of the NFBSI-16 in clinical studies. Currently, the longitudinal validity of the web-based version of the NFBSI-16 and the validity of several other demographic groups in China are being investigated, giving clinicians more choice when evaluating health-related symptoms and quality of life in patients with breast cancer and other malignant tumors.

## Appendix

Multimedia Appendix 1 Screenshot of web-based version National Comprehensive Cancer Network–Functional Assessment of Cancer Therapy–Breast Cancer Symptom Index (NFBSI-16) questionnaire.

## Abbreviations

ANOVA: analysis of variance
CCC: concordance correlation coefficient
DRS-E: Disease-Related Symptoms Subscale–Emotional
DRS-P: Disease-Related Symptoms Subscale–Physical
FACIT: Functional Assessment of Chronic Illness Therapy
FWB: Function and Well-Being Subscale
ICC: intraclass correlation coefficient
NFBSI-16: National Comprehensive Cancer Network–Functional Assessment of Cancer Therapy–Breast Cancer Symptom Index
PROMs: patient-reported outcome measures
TSE: Treatment Side Effects Subscale
