# Supplementary figures and images for: Comparative Analysis of Paper-Based and Web-Based Versions of the National Comprehensive Cancer Network-Functional Assessment of Cancer Therapy-Breast Cancer Symptom Index (NFBSI-16) Questionnaire in Breast Cancer Patients: Randomized Crossover Study
Source: JMIR Med Inform. 2021 Mar 2;9(3):e18269. doi: 10.2196/18269 (PMC7967224; doi:10.2196/18269)

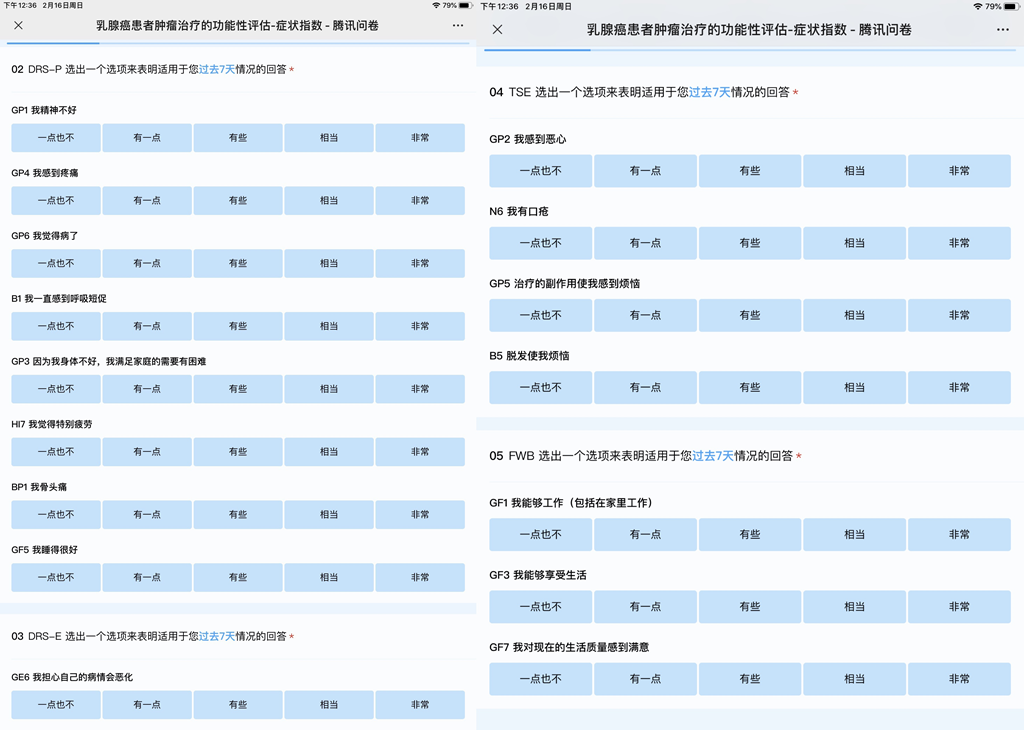

Supplement: Multimedia Appendix 1 [file medinform_v9i3e18269_app1.png]
